# Monitoring data of the Høje Taastrup water pit thermal energy storage

**DOI:** 10.1016/j.dib.2025.111305

**Published:** 2025-01-14

**Authors:** Ioannis Sifnaios, Simon Furbo, Adam R. Jensen

**Affiliations:** Department of Civil and Mechanical Engineering, Technical University of Denmark, Kgs. Lyngby, Denmark

**Keywords:** Heat storage, Short-term storage, Measurement data, PTES

## Abstract

The pit thermal energy storage (PTES) in Høje Taastrup, Denmark, was the first large-scale PTES to be operated as a short-term storage (storage cycle of 1-2 weeks). The storage was connected to the Copenhagen district heating grid and started operating in February 2023. In addition to the unique use case, the storage represents the state-of-the-art PTES system, featuring an innovative lid construction and a custom-developed polymer liner. Monitoring data of the storage operation are provided freely for 2024, including measurements of the storage water temperature, charged/discharged energy, diffuser flow rates and temperatures, lid heat flux, humidity, and temperatures, soil temperature, and ambient conditions. The dataset can be used to assess the storage performance and, more importantly, validate simulation models, which has not been done for short-term PTES systems. The data is freely available on GitHub.

Specifications Table

**Table:** 

Subject	Energy Engineering and Power Technology
Specific subject area	Large-scale thermal energy storage operated for district heating
Type of data	Raw measurement data and metadata from January to September 2024
Data collection	Data was collected by a Supervisory Control and Data Acquisition (SCADA) system and saved on an FTP server
Data source location	Høje Taastrup, Denmark (latitude: 55.666°N, longitude: 12.252°E)
Data accessibility	Data can be freely downloaded from the GitHub repository: https://github.com/PitStorages/HojeTaastrupData Zenodo DOI: 10.5281/zenodo.14050124
Related research article	None

## 1 Value of the Data

• This dataset provides detailed insights into the operation and performance of the first large short-term pit thermal energy storage (PTES) system. The data allows for validating and improving simulation models for short-term and long-term PTES operations, which previously relied only on data from seasonal storage systems.
• Additionally, the data can also be used to enhance the accuracy and reliability of simulation tools for PTES, for example, for software such as TRNSYS, Modelica, or custom PTES models.
• With only one other open-access PTES dataset available [1], this dataset addresses the severe lack of openly accessible data in this field. Open access ensures that a wide range of stakeholders (e.g., researchers, consultants, energy companies, and non-profit organizations) can utilize these data without barriers, promoting innovation, collaboration, and transparency within the industry.
• The main target group is engineers, consultants, and energy planners designing district heating systems and energy storage solutions, who will benefit from the dataset as it provides real-world data that can be used to support feasibility studies and system optimization.
• This data article provides a thorough description of the storage and accompanying metadata, providing users with sufficient information for maximizing the utility of the data.

## 2 Background

**Fig. 1:**
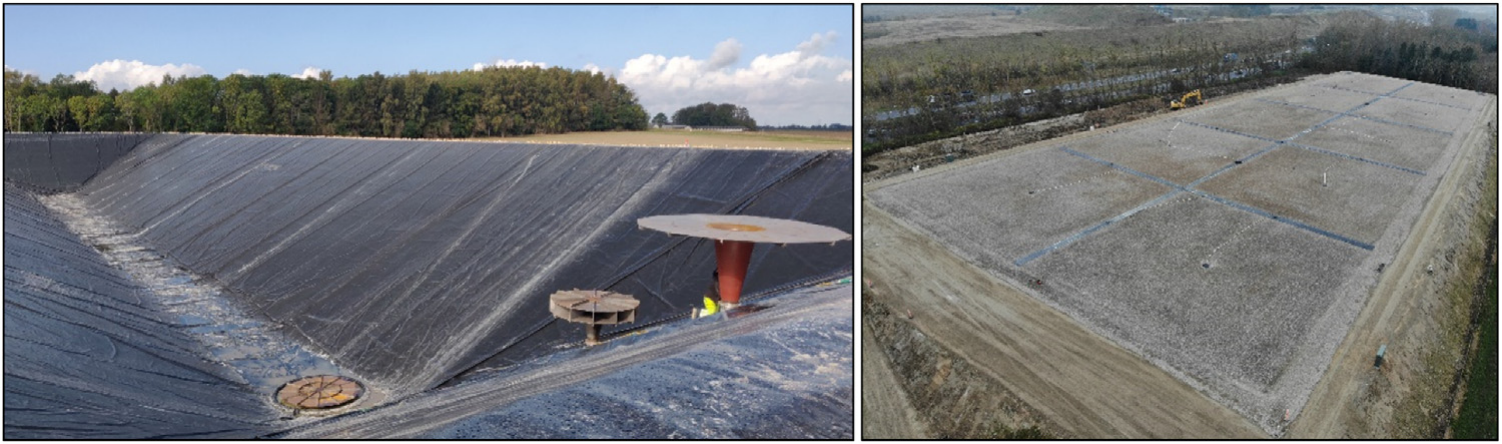
Construction (left) and final view (right) of the PTES in Høje Taastrup.

In 2023, the first large-scale, short-term operated pit thermal energy storage (PTES) was constructed in Høje Taastrup, Denmark, with a volume of 70,000 m^3^. A PTES is a large water reservoir dug into the ground, lined with a watertight polymer liner (to prevent water from leaking to the ground), and covered with a floating insulating lid to reduce heat losses [1]. The storage is used to store hot water (upper limit of 90°C), which is used for district heating. An overview of the PTES is presented in Fig. 1.

Historically, PTES systems have mainly been used for seasonal heat storage. Most of these storages are located in Denmark: Dronninglund (60,000 m^3^) [2], Marstal (75,000 m^3^) [3], Gram (122,000 m^3^) [4], Vojens (200,000 m^3^) [5], and Toftlund (70,000 m^3^) [6]. The only short-term PTES was in Tibet; however, this storage was fairly small (15,000 m^3^), and data from its operation are not available. Consequently, the scientific community has shown a great interest in the PTES in Høje Taastrup. Since this PTES was constructed as part of the FLEX-TES research project [7], documentation and monitoring data can be made publicly available. Detailed information on the design and construction of the storage is available in [8].

## 3 Data Description

The data associated with this article are distributed in a CSV file named “ptes_operation_data_hoje_taastrup_2024.csv”, which contains the monitoring data from the Høje Taastrup PTES for 2024.

**Fig. 2:**
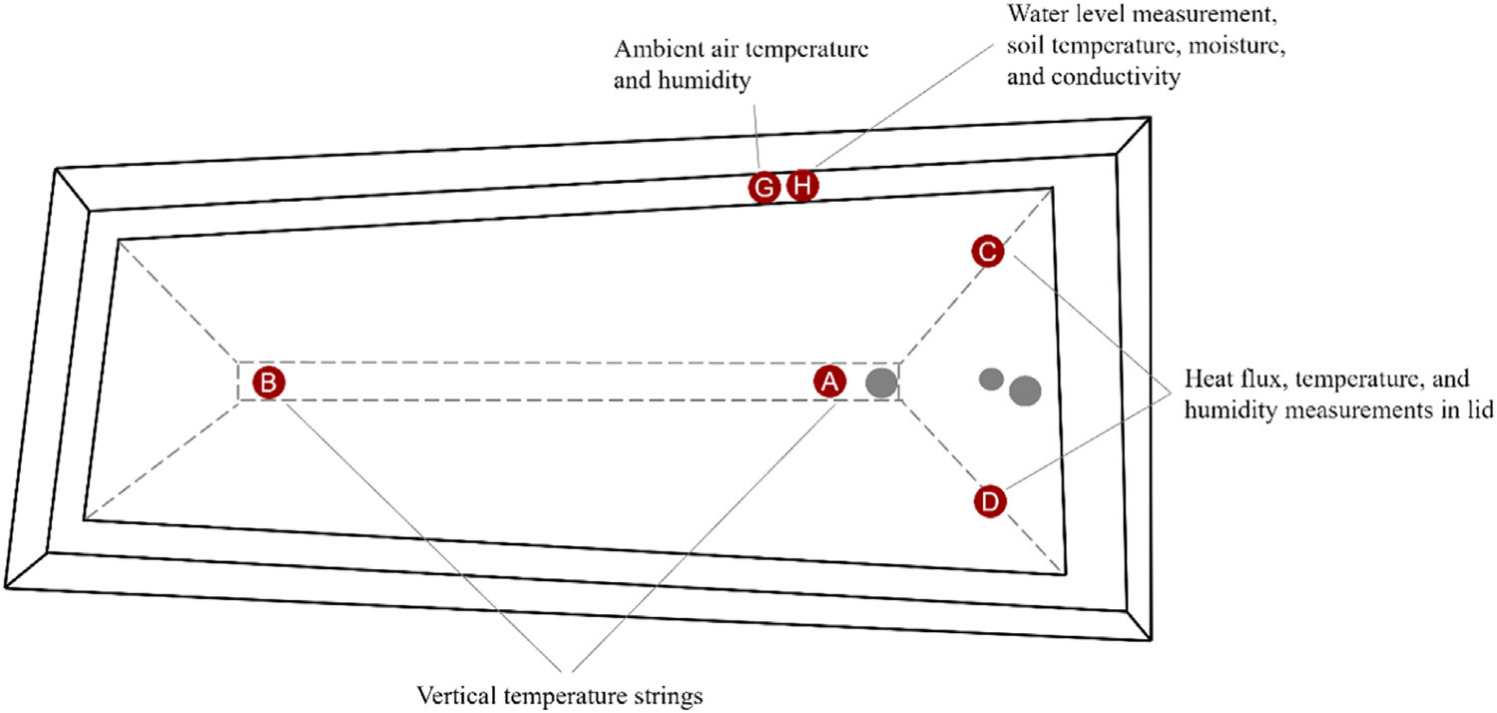
Top view of the installed measurement equipment and their locations.

**Fig. 3:**
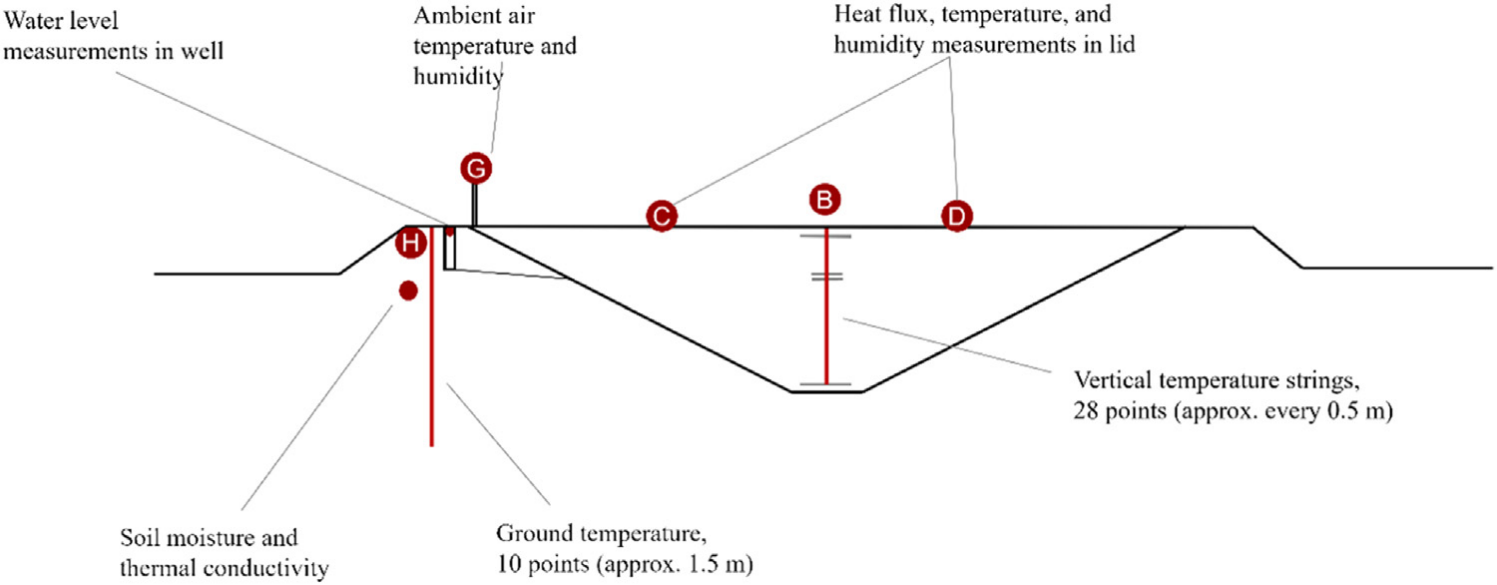
Side view of the installed measurement equipment and their locations.

Table 1 presents an overview of the sensors and available metadata, including the sensor type, units, accuracy, and the tag names used in the dataset. Fig. 2, Fig. 3 show a top and side view of the installed sensors.

**Table 1:** Metadata of the available sensors.

Tag name	Description	Sensor	Unit	Uncertainty
A_00.25m	Water temperature at location A (0.25m under the lid)	Temperature sensor (PT100), Krohne MP-14xW10-R330	°C	0.15 K (0°C), 0.35 K (100°C)
⋮	⋮	⋮	⋮	⋮
A_13.80m*	Water temperature at location A (13.8m under the lid)	Temperature sensor (PT100), Krohne MP-14xW10-R330	°C	0.15 K (0°C), 0.35 K (100°C)
B_00.25m	Water temperature at location B (0.25m under the lid)	Temperature sensor (PT100), Krohne MP-14xW10-R330	°C	0.15 K (0°C), 0.35 K (100°C)
⋮	⋮	⋮	⋮	⋮
B_11.25m	Water temperature at location B (11.25m under the lid)	Temperature sensor (PT100), Krohne MP-14xW10-R330	°C	0.15 K (0°C), 0.35 K (100°C)
T_ground_1.30m	Ground temperature at a depth of 1.3 m under the embankment	Temperature sensor (PT100), Krohne MP-14xW10-R330	°C	0.15 K (0°C), 0.35 K (100°C)
⋮	⋮	⋮	⋮	⋮
T_ground_15.00m	Ground temperature at a depth of 15 m under the embankment	Temperature sensor (PT100), Krohne MP-14xW10-R330	°C	0.15 K (0°C), 0.35 K (100°C)
T_lid_1_1	Temperature in the lid in location C, position 1 (bottom of the lid, close to the water)	Temperature sensor (PT100), Krohne OPTITEMP TRA-W10	°C	0.15 K (0°C), 0.35 K (100°C)
⋮	⋮	⋮	⋮	⋮
T_lid_1_5	Temperature in the lid in location C, position 5 (top of the lid, close to the ambient air)	Temperature sensor (PT100), Krohne OPTITEMP TRA-W10	°C	0.15 K (0°C), 0.35 K (100°C)
T_lid_2_1	Temperature in the lid in location D, position 1 (bottom of the lid, close to the water)	Temperature sensor (PT100), Krohne OPTITEMP TRA-W10	°C	0.15 K (0°C), 0.35 K (100°C)
⋮	⋮	⋮	⋮	⋮
T_lid_2_5	Temperature in the lid in location D, position 5 (top of the lid, close to the ambient air)	Temperature sensor (PT100), Krohne OPTITEMP TRA-W10	°C	0.15 K (0°C), 0.35 K (100°C)
lid_humidity_1_1	Relative humidity in the lid location C, position 1 (bottom of the lid, close to the water)	Hygrosmart HS3 probe	%R.H.	1 % R.H.
lid_humidity_1_2	Relative humidity in the lid location C, position 2 (top of the lid, close to the ambient air)	Hygrosmart HS3 probe	%R.H.	1 % R.H.
lid_humidity_2_1	Relative humidity in the lid location D, position 1 (bottom of the lid, close to the water)	Hygrosmart HS3 probe	%R.H.	1 % R.H.
lid_humidity_2_2	Relative humidity in the lid location D, position 2 (top of the lid, close to the ambient air)	Hygrosmart HS3 probe	%R.H.	1 % R.H.
F_top	Flow rate in the top diffuser	Ultrasonic flow meter, Krohne Optisonic 3400	m³/hr	0.3 % + 0.6 m³/hr
F_mid	Flow rate in the middle diffuser	Ultrasonic flow meter, Krohne Optisonic 3400	m³/hr	0.3 % + 0.6 m³/hr
F_bot	Flow rate in the bottom diffuser	Ultrasonic flow meter, Krohne Optisonic 3400	m³/hr	0.3 % + 0.6 m³/hr
T_top	Water temperature in the top diffuser	Temperature sensor (PT100), Krohne Optitemp TRA-T30	°C	0.15 K (0°C), 0.35 K (100°C)
T_mid	Water temperature in the middle diffuser	Temperature sensor (PT100), Krohne Optitemp TRA-T30	°C	0.15 K (0°C), 0.35 K (100°C)
T_bot	Water temperature in the bottom diffuser	Temperature sensor (PT100), Krohne Optitemp TRA-T30	°C	0.15 K (0°C), 0.35 K (100°C)
T_bot_0.2m*	Water temperature at 0.2 m from the bottom of the storage	Temperature sensor (PT100), Krohne Optitemp TRA-W80	°C	0.15 K (0°C), 0.35 K (100°C)
T_bot_1.2m	Water temperature at 1.2 m from the bottom of the storage	Temperature sensor (PT100), Krohne Optitemp TRA-W80	°C	0.15 K (0°C), 0.35 K (100°C)
T_bot_2.2m	Water temperature at 2.2 m from the bottom of the storage	Temperature sensor (PT100), Krohne Optitemp TRA-W80	°C	0.15 K (0°C), 0.35 K (100°C)
Heat_flux_1	Heat flux through lid at position C	Hukseflux HFP01	W/m²	∼20 %
Heat_flux_2	Heat flux through lid at position D	Hukseflux HFP01	W/m²	∼20 %
E_charge	Energy charged into the PTES	Calculated value	MWh	-
E_discharge	Energy discharged into the PTES	Calculated value	MWh	-
T_amb	Ambient air temperature	Luft WS601-UMB Smart Weather Sensor	°C	0.2 K
Air_humidity	Relative air humidity	Luft WS601-UMB Smart Weather Sensor	%R.H.	2 % R.H.
Wind_speed	Wind speed	Luft WS601-UMB Smart Weather Sensor	m/s	0.3 m/s
Guided_radar_level	PTES water level measured with a guided radar sensor	Krohne Optiflex 7200C	m amsl	2 mm
Pressure_level	PTES water level measured with a pressure level transmitter sensor	Klay 2000-Hydrobar EXTD	m amsl	3.6 mm

**Note**: A_13.80m and T_bot_0.2m are the same sensor. They are included in the A strings to calculate the internal energy of the PTES.

## 4 Experimental Design, Materials and Methods

### 4.1 Storage description

The FLEX-TES research project [7] started in 2018 and will be completed in 2025. Part of the project was to design and construct a short-term PTES in Høje Taastrup, as well as to monitor its operation for two years. The main aim was to prove the application of PTES for short-term storage, thus unlocking a new market for PTES systems and establishing PTES as a key solution to integrate fluctuating renewable energy sources into DH systems. Furthermore, the project aimed to demonstrate that the temperature in the upper part of the storage could be consistently maintained at 90°C, positioning PTES as a cost-effective alternative to more expensive steel tanks. This was achieved through advances in liner technology, insulation materials, and system design [8].

The developed PTES was jointly owned by the companies VEKS and Høje Taastrup Fjernvarme a.m.b.a. VEKS is a transmission company that provides district heating to 17 distribution companies in the western part of Copenhagen. Høje Taastrup Fjernvarme a.m.b.a. is a consumer-owned distribution company delivering district heating in the municipality of Høje Taastrup. Notably, the expenses related to the construction and operation of the storage were shared among all of the major heat producers in the Copenhagen DH network according to their individual expected long-term economic benefits.

### 4.2 Storage design and dimensions

**Fig. 4:**
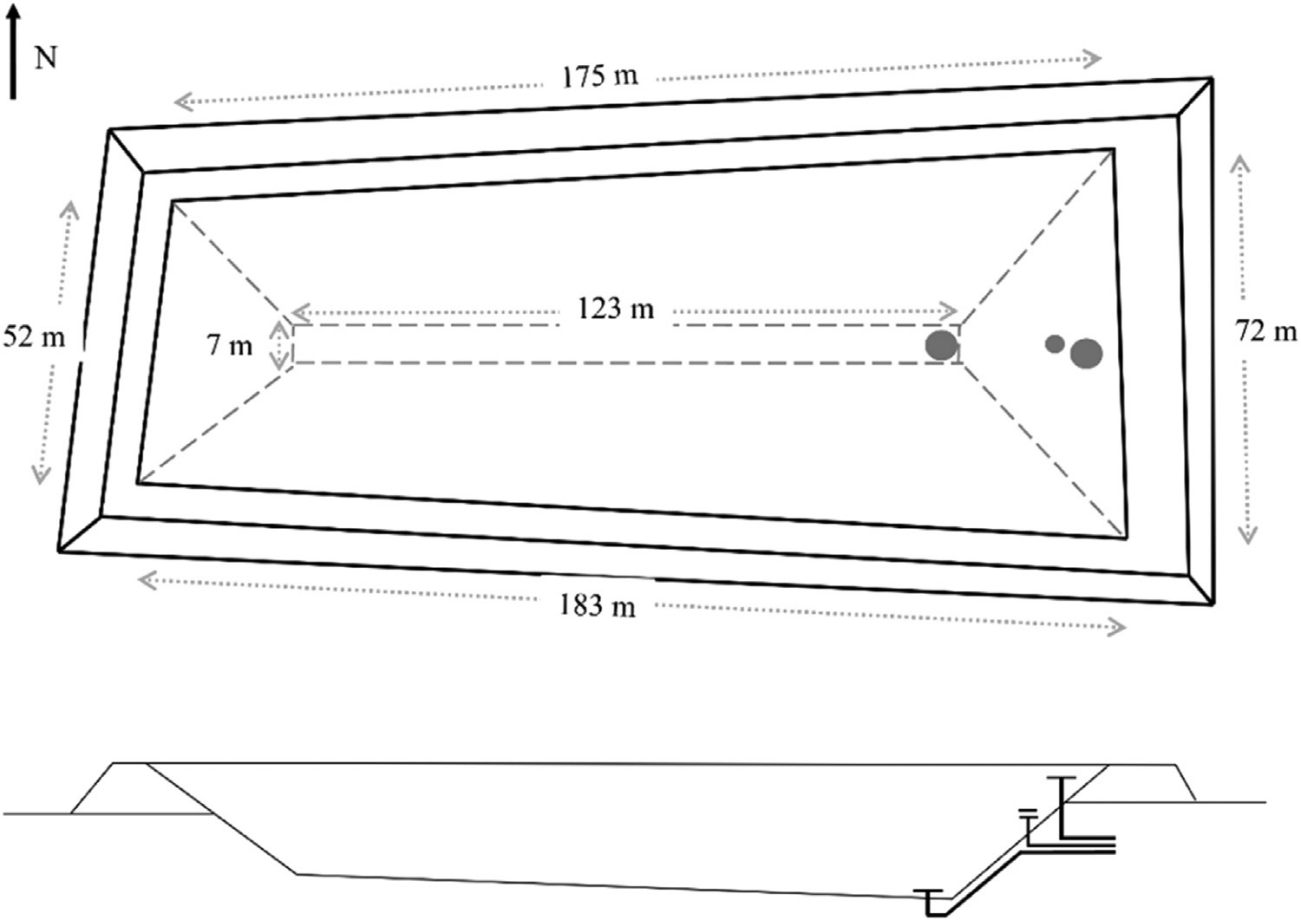
Schematic of the storage dimensions.

The shape and dimensions of the PTES are presented in Fig. 4. It can be observed that the shape of the PTES is irregular, unlike existing systems, which are usually shaped as a truncated pyramid/obelisk. The irregular shape of the PTES was the result of the following requirements and limitations:

• The available land plot was limited on the north side by a drinking water pipe and on the south side by a highway.
• The storage construction followed the general practice of compacting the excavated soil to form embankments around the storage. The steepness of the embankments was dictated by the soil stability and the facilitation of in situ liner welding. Thus, the PTES sides had a slope between 1:2 and 1:2.1.

It should be noted that the above limitations also led to a slanted storage bottom (see Fig. 4). The reason was that if the west side of the PTES was deeper, there would not be a horizontal part at the bottom, and liner welding would not be possible. Consequently, the deepest point of the PTES (east) had a depth of 14.05 m, while the shallowest point (west) had a depth of 12 m. The constructed PTES had a volume of approximately 70880 m^3^ with a lid area of 11108 m^2^ and a bottom area of 864 m^2^.

The three diffusers were placed at different heights from the bottom of the storage: the top diffuser at 13.8 m, the middle diffuser at 10.5 m, and the bottom diffuser at 0.45 m. These heights correspond to the bottom diffuser plate for the top and middle diffusers and to the top diffuser plate for the bottom diffuser. It should be noted that the middle diffuser was placed so that approximately half of the volume was above it and half below it.

### 4.3 Soil properties

The thermal properties of the soil tests were carried out following the ASTM D 5334:14 [9], and were measured in late May 2020, after the pit had been excavated and before the liner installation. The properties were measured using a TEMPOS Thermal Properties Analyzer by METER Group [10]. The TEMPOS instrument uses the "transient line heat source method" for measuring the thermal conductivity of porous materials. The probe for this type of measurement consists of a needle with a heating element and temperature sensor inside. A current is passed through the heating element, and the system monitors the sensor's temperature over time. The thermal conductivity can then be estimated from the time dependence of the sensor temperature while the probe is inserted into the material under test. It is also possible to have a dual-probe sensor, where the heating element and temperature sensor are in separate needles. The benefit of the dual-probe sensor is that the analysis of the temperature versus time relationship for the separated probes also yields information on diffusivity and heat capacity, in addition to conductivity. The accuracy of the TEMPOS device for thermal conductivity, diffusivity, and heat capacity measurement is 10%.

Soil samples were taken to measure the density and water content of the soil. The samples were taken using cylindrical metal containers with a height of 140 mm and a radius of 21 mm. The cylinders were used to extract soil samples, which were weighted on-site using a scale with an accuracy of ± 1 gram. Since the inner volume of the cylinder was easy to calculate, the bulk density of the soil was found by dividing the weight of the soil by its volume.

The water content of the soil samples was determined by weighing the undisturbed soil samples and then heating them in an oven for 48 hours. After the water had evaporated from the soil, each sample was weighed again to calculate the change in weight. The water content of the soil was found to be approximately 10%.

In order for the obtained results to be as accurate as possible and independent of the location in the pit storage, samples were taken from 11 different locations. Thermal conductivity, thermal diffusivity, and volumetric heat capacity were measured at all sampling locations, and multiple measurements were made at each location. The average soil thermal properties and their standard deviation are reported in Table 2.

**Table 2:** Measured soil properties.

Parameter	Mean value	Std. Dev.	No. samples	Unit
Thermal conductivity	2.23	0.32	82	W/(m K)
Thermal diffusivity	0.87	0.21	38	mm^2^/s
Volumetric heat capacity	2.71	0.46	38	MJ/(m^3^ K)
Bulk density	2130	145	11	kg/m^3^
Water content	9.6	0.9	4	% w/w

According to the soil investigation report conducted by the company GEO, the soil type at a depth of 14 m was classified as clay till: very sandy, gravelly, and grey. However, it was observed that due to differences in the sand concentration, the soil could look more grey (more clay content) or more brown (more sand content). The two different colors of soil were also found to have minor differences in density due to the lower density of the sand. It should be mentioned that these values correspond to the soil conditions on the day of the measurements. These results could change according to the water content of the soil, which could be caused by seasonal variations in precipitation, leakage of water from the PTES to the soil, and/or drying of soil due to the PTES operation.

### 4.4 Storage operation

The PTES was charged by the main DH transmission grid (which is operated at 110°C) and discharged to the local distribution grid via heat exchangers. Charging and discharging are done using three diffusers located at the storage's top, middle, and bottom (see Fig. 4).

During charging, hot water was supplied to the PTES using the top diffuser, while cold water was extracted using the bottom diffuser. The storage water temperature was limited to a maximum of 90°C due to the limitations of the liner material. Discharging can be done in three different modes:

1. Hot water is extracted from the PTES using only the top diffuser, while cold water enters the storage using the bottom diffuser
2. Hot water is extracted from the PTES using both the top and middle diffuser, while cold water enters the storage using the bottom diffuser
3. Hot water is extracted from the PTES using only the middle diffuser, while cold water enters the storage using the bottom diffuser

**Fig. 5:**
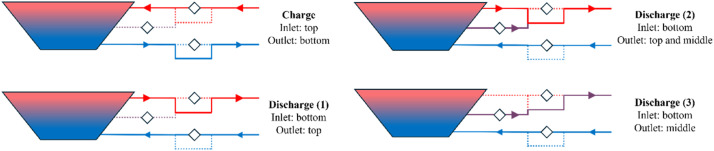
Schematic of the utilization of the diffusers for the different operation modes. Solid lines represent pipes in operation for the specific mode, whereas dashed lines are not in operation. The rotated square symbols represent the locations of the flow meters.

Essentially, operating the middle diffuser during discharge enables water discharge at a lower temperature by mixing it with hotter water from the top diffuser. It should be noted that the storage could also be discharged using only the top diffuser; thus, the middle diffuser adds flexibility but is not essential for operation. A schematic of the charge/discharge modes is presented in Fig. 5.

It should be noted that although one flow meter was installed at each pipe leading to a diffuser, not all flow meters are used simultaneously. As can be seen in Fig. 5, some flow meters are bypassed during the charge and discharge operations. Essentially, only the flow meter at the pipe where water comes into the storage is used, and possibly the flow meter of the middle diffuser if the middle diffuser is in operation. In general, it would have been preferred to have the flow meters installed such that all flow meters were always used.

### 4.5 Installed sensors

In order to monitor the operation and performance of the PTES in Høje Taastrup, a number of sensors were installed. The specific location of the sensors can be seen in Fig. 2, Fig. 3. The monitoring data include the following parameters:

• Water temperature
• Storage water level
• Lid conditions (temperature, humidity, and heat flux)
• Ground temperature
• Ambient conditions (air temperature, wind speed, and humidity)
• Diffuser temperature and flow rate
• Charged/discharged energy

Each one of these parameters is described in detail in the sections below. It should be noted that sensors were also installed for measuring soil humidity, soil thermal conductivity, and precipitation. However, these sensors were not included in the dataset due to installation issues.

### 4.6 Water temperature

The storage water temperature was measured using four temperature strings, each having 14 PT100 sensors. Two strings were placed at the east end of the PTES and two at the west end (locations A and B in Fig. 2). A vertical offset between the sensor locations in the two temperature strings ensured a better vertical resolution. It should be noted that the sensors were spaced closer together at the top and bottom of the storage (every 0.25 m), whereas the distance between sensors was greater in the middle (every 0.5 m). The water temperature was measured at both the east and west ends to investigate the storage's horizontal temperature profile. Specifically, it was of interest to investigate whether the heat could be transported to and from the western end of the storage, given that the inlets/outlets were at the eastern end.

### 4.7 Storage water level

The PTES lid was flexible and moved up and down to accommodate the change in water level due to the change in density caused by variations in the water temperature. However, the lid can be damaged if the water level is too high or too low. Thus, it is important to add water to the storage when the water level drops in order to ensure the safety of the lid construction. Consequently, two water level sensors were installed in a small well connected to the PTES in order to monitor the water level in the storage. The two sensors used different measurement technologies (i.e., guided radar and hydrostatic pressure). The SCADA system was configured to raise an alarm when the water level dropped below a certain threshold. The water level measurements are in m above mean sea level (m amsl). The deepest part of the storage is the eastern end, which is 22.3 m amsl.

It should be noted that the measured water level in the well is approximately 10 cm higher than the actual water level under the lid. This difference occurs due to the lid's weight, which is approximately 100 kg/m^2^.

### 4.8 Lid conditions

**Fig. 6:**
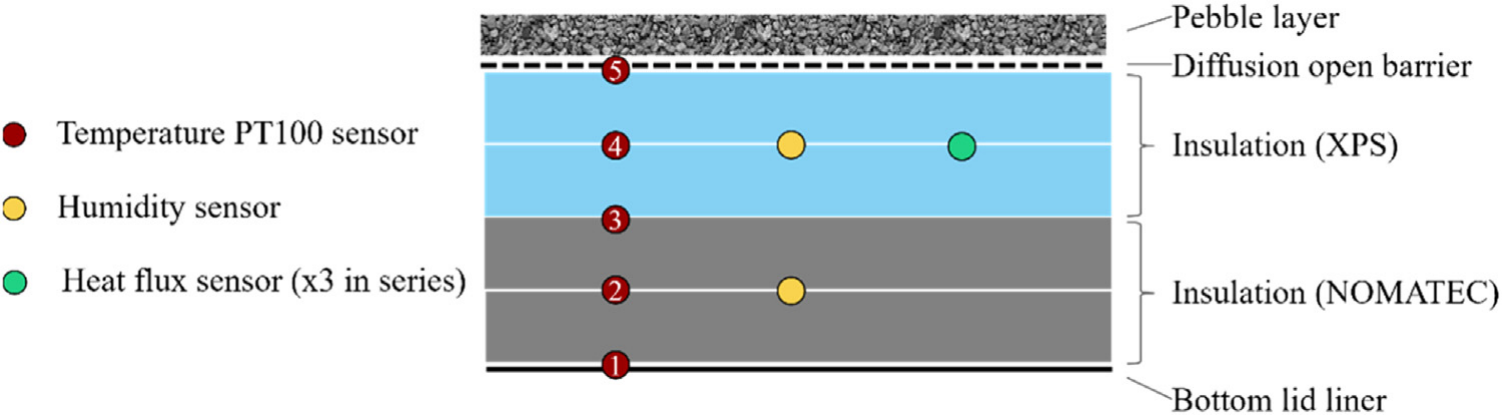
Cross section of the PTES lid with the locations of the installed sensors.

The lid consisted of two XPS insulation layers and two NOMATEC insulation layers sandwiched between a PP liner and a diffusion barrier. Five temperature, two humidity, and three heat flux sensors were installed in the lid at two different locations (C and D in Fig. 2). In order to increase the measuring accuracy of the heat flux sensors, the three heat flux sensors were connected in series at each measurement location. The temperature and humidity sensors were placed between the insulation layers, as shown in Fig. 6.

The thermal characteristics of the insulation layers specified by the manufacturer were:

• NOMATEC foam mats with a thickness of 0.0625 m per layer, with a thermal conductivity of 0.0483 W/(m K) at 82°C and 0.0462 W/(m K) at 66°C.
• XPS polystyrene boards with a thickness of 0.075 m per layer, with a thermal conductivity of 0.0397 W/(m K) at 46°C and 0.0358 W/(m K) at 22°C.

Using this information, the theoretical U-value of the entire lid was calculated to be approximately 0.151 W/(m^2^ K), although this depends on the ambient temperature and the water temperature at the top of the PTES.

### 4.9 Ground temperature

The ground temperature was measured using a string of 11 temperature sensors (PT100) buried in the embankment (see Fig. 3). The sensors were placed approximately 4 m from the water edge and were located near the northeast corner of the storage (see Fig. 2). There was a 1.4 m offset between the sensors, reaching a depth of 15 m from the top of the embankment.

### 4.10 Ambient conditions

A Luft WS601-UMB weather station was mounted on a 5 m-high pole on the storage embankment and was used to measure ambient air temperature, humidity, and wind speed. These measurements were used to investigate the heat losses through the lid and to monitor the weather conditions around the PTES. It should be noted that weather measurements can also be obtained from the Danish Meteorological Institute (DMI), which operates a weather station (06170 Roskilde airport) approximately 12 km southwest of the storage.

### 4.11 Diffuser temperature and flow rate

As previously mentioned, only two of the three flow meters measure at the same time:

• Only the top flow measured: charge scenario
• Only the bottom flow measured: discharge scenario (1)
• Only the bottom and middle flow is measured: discharge scenarios (2) and (3)

In order to better understand the operation and use the data for simulations, it is helpful to distinguish the flow rates as positive (when water enters the storage) and negative (when water exits the storage). It should be noted that using this convention, the flow rate of the middle diffuser will always be negative.

For simulation models, it is important that there is flow balance in the storage (i.e., the same amount of water enters and exits the storage during charge and discharge operations). Thus, flow balance was used to calculate the missing diffuser flow rates as presented in the data treatment script on GitHub.

### 4.12 Charged/discharged energy and efficiency

It should be noted that the charged and discharged energy provided in the dataset are the calculated values using the flow rates, diffuser temperatures, and temperature-dependent functions for water density and specific heat. Additionally, since there are a number of ways to estimate the storage efficiency, the monthly charged/discharged energy and energy efficiency are provided in Table 3 as a reference. The expression suggested in [11] was used to calculate the efficiency. For information on the PTES performance in 2023, the reader is referred to [12].

**Table 3:** Charged and discharged energy and energy efficiency for 2024.

Month	Charged energy [MWh]	Discharged energy [MWh]	Internal energy change [MWh]	Efficiency [%]
January	1396	1629	-434	89
February	2961	2297	215	84
March	4993	4307	268	91
April	3793	3901	-322	95
May	3613	2161	994	83
June	1362	2112	-894	94
July	2660	1299	960	76
August	768	1534	-980	88
September	1388	1870	-641	92
Total	22934	21109	-833	89

### 4.13 Quality control and handling of missing data

The quality control method described in [1] was used to screen and remove erroneous data in the dataset. For example, water temperatures and air relative humidity values outside the range 0-100°C were filtered out, as well as unfeasible spikes. However, it should be noted that this treatment was not necessary for most sensors as there were very few periods with erroneous data. For specific details, the reader is referred to the data treatment script on GitHub, where all data treatment is presented.

## Limitations

Not applicable.

## Ethics Statement

The authors have read and followed the ethical requirements for publication in Data in Brief and confirm that the current work does not involve human subjects, animal experiments, or any data collected from social media platforms

## Credit Author Statement

**Ioannis Sifnaios:** Conceptualization, Data curation, Visualization, Writing, Original draft preparation. **Simon Furbo:** Supervision, Funding acquisition, Writing- Reviewing and Editing. **Adam R. Jensen:** Conceptualization, Data curation, Writing- Reviewing and Editing.

## Data Availability

Zenodoptes_operation_data_hoje_taastrup_2024 (Original data).
